# 28.2 DISORDERS OF THE EMBODIED SELF IN SCHIZOPHRENIA: AT THE CROSSROAD BETWEEN DEVELOPMENT AND PSYCHOPATHOLOGY

**DOI:** 10.1093/schbul/sby014.117

**Published:** 2018-04-01

**Authors:** Andrea Raballo, Michele Poletti

**Affiliations:** 1 Norwegian University of Science and Technology; 2 Department of Mental Health Reggio Emilia

## Abstract

**Background:**

Basic disorders of the embodied self (BDES) encompass a cloud of related clinical constructs (e.g. cenesthesias, distortions of somato-psychic unity, anomalous bodily experiences in a broad sense) that are immanently related to a profound transformation of subjectivity and with the developmental modulation of bodily awareness. They have been historically ascribed a potential role in the emergence of schizophrenia spectrum disorders. However, the clinical-phenomenological level of description has been only marginally integrated with novel insights from developmental psychopathology and neurosciences. ​

**Methods:**

We conducted a conceptual literature review based on clinical analysis and heuristic synthesis.

**Results:**

Despite often occurring in prodromal/clinical at-risk states, as well as in full blown schizophrenia spectrum conditions (where BDES play a pivotal psychopathogenetic role in the genesis of productive symptoms), they are relatively neglected both in research and in routine clinical examination. Furthermore, BDES also discriminate non help-seeking genetic high risk subjects from normal controls.

**Discussion:**

Within the superordinate construct of Self-disorders, BDES are a potentially relevant dimensional phenotype for the characterization of broad Schizophrenia Spectrum vulnerability. Their contextualization within a developmental and neurophysiological perspective could further amplify their value for etio-pathogenetic research. ​

